# Epigenetic Mechanisms in Autoimmune Thyroid Diseases: Bridging Research and Clinical Applications

**DOI:** 10.3390/ijms262411823

**Published:** 2025-12-07

**Authors:** Shouxia Xiao, Yuelin Hu, Xin Wang, Hongsong Yu

**Affiliations:** 1 Department of Immunology, Special Key Laboratory of Gene Detection and Therapy of Guizhou Province, Zunyi Medical University, Zunyi 563000, China; xiaoshouxia@zmu.edu.cn (S.X.); huyuelin@zmu.edu.cn (Y.H.); 2 School of Basic Medical Sciences, Key Laboratory of Cancer Prevention and Treatment of Guizhou Province, Zunyi Medical University, Zunyi 563000, China; wangxin@zmu.edu.cn

**Keywords:** autoimmune thyroid disease, Graves’ disease, Hashimoto’s thyroiditis, DNA methylation, histone modification, non-coding RNA

## Abstract

Autoimmune thyroid disease (AITD) exemplifies an organ-specific autoimmune disorder, including Hashimoto’s thyroiditis (HT) and Graves’ disease (GD). HT is characterized by hypothyroidism, whereas GD primarily presents as hyperthyroidism. Immunological evidence indicates that AITD pathogenesis requires both a permissive genetic background and environmental triggers to initiate and sustain disease progression. However, the exact molecular and cellular pathways through which these elements synergize to trigger and sustain autoimmune responses remain unclear. Emerging evidence suggests that epigenetic regulation serves as the key interface decoding genetic predisposition through environmental stimuli in AITD etiology. Studies show that environmental epigenetic reprogramming initiates AITD development in genetically susceptible individuals. Epigenetic regulators, including DNA methylation, histone modifications, and non-coding RNA activity, finely tune transcriptional outputs to influence disease trajectories. Beyond elucidating AITD pathogenesis, these epigenetic alterations offer clinical value as diagnostic biomarkers and modifiable therapeutic targets, facilitating precision medicine approaches from early detection to customized interventions. These epigenetic modifications not only elucidate AITD pathophysiology but also provide measurable markers for early diagnosis and molecular targets for personalized treatment strategies.

## 1. Introduction

Autoimmune thyroid disease (AITD) occurs when the immune system targets thyroid antigens, leading to progressive tissue damage and functional impairment. AITD primarily manifests as either Hashimoto’s thyroiditis (HT) or Graves’ disease (GD) [1]. HT is characterized by significant lymphocytic infiltration and destruction of thyroid tissue, resulting in hypothyroidism [2]. Patients with HT often experience symptoms such as fatigue, weight gain, cold intolerance, myxedema, and cognitive impairment [3,4,5]. Conversely, GD is marked by the presence of stimulating autoantibodies that cause excessive activity of the thyroid gland, leading to hyperthyroidism [6,7]. This condition typically presents with symptoms like unintended weight loss, heat intolerance, tachycardia, tremors, and increased anxiety [8]. Despite their contrasting clinical manifestations, GD and HT share a common immunological basis involving the breakdown of self-tolerance to thyroid antigens through distinct dysregulatory pathways. When immune tolerance breaks down, thyroid-specific autoantigens become immunogenic targets, triggering the production of pathogenic autoantibodies including thyroid peroxidase antibodies (TPOAb) and thyroglobulin antibodies (TGAb) [9,10]. These autoantibodies mediate thyroid-specific cytotoxicity, causing structural damage and functional impairment of thyroid follicular cells. As a result, thyroid hormone synthesis and secretion are disrupted, histopathological alterations occur in thyroid tissue, and diverse clinical features characteristic of AITD emerge [11].

Current therapeutic strategies for AITD primarily rely on pharmacological approaches. A randomized trial conducted by Xie et al. [12] demonstrated that a combination of low-dose methotrexate and methimazole for treating GD resulted in a higher rate of medication discontinuation and a faster decline in thyrotropin-related antibodies at 18 months, with a safety profile comparable to methimazole alone. Although pharmacotherapy is commonly used in managing AITD, it often causes significant adverse effects such as urticaria and thrombocytopenia. Additionally, disease recurrence remains a considerable challenge despite adequate symptom control [13]. Radioactive iodine (^131^I) therapy, which utilizes its strong β-emission and tissue-specific radiation absorption, poses significant iatrogenic risks. These include acute complications such as sialadenitis, nasolacrimal duct obstruction, and gonadal dysfunction, as well as hematopoietic suppression. Furthermore, delayed adverse outcomes include persistent hypothyroidism and secondary malignancies [14,15]. Thyroidectomy, while clinically indicated in certain cases, invariably results in permanent hypothyroidism requiring lifelong hormone supplementation. This procedure also carries surgical risks including hypoparathyroidism and recurrent laryngeal nerve damage, potentially leading to chronic complications [16]. The current management of AITD faces significant therapeutic challenges, highlighting the critical need for deeper investigation into disease mechanisms to develop targeted therapies with improved safety and efficacy profiles.

The precise pathogenic mechanisms underlying AITD remain incompletely understood, reflecting the intricate interactions among genetic susceptibility, environmental factors, and immune system dysfunction. Although dysregulated immune responses are the primary pathogenic driver, genetic susceptibility and environmental triggers are increasingly recognized as critical modulators of AITD progression [17]. Emerging evidence suggests that environmental factors interact with genetic susceptibility loci, creating synergistic effects mediated through epigenetic regulatory pathways in AITD pathogenesis [18]. Epigenetic regulation involves heritable modifications that dynamically control gene expression patterns through DNA methylation, histone modifications, and non-coding RNAs, while preserving the primary nucleotide sequence. These processes form an integrated epigenetic framework for precise transcriptional regulation. Disease-specific epigenetic alterations in AITD pathogenesis reveal novel therapeutic opportunities and potential diagnostic biomarkers [19,20,21].

**Figure 1:**
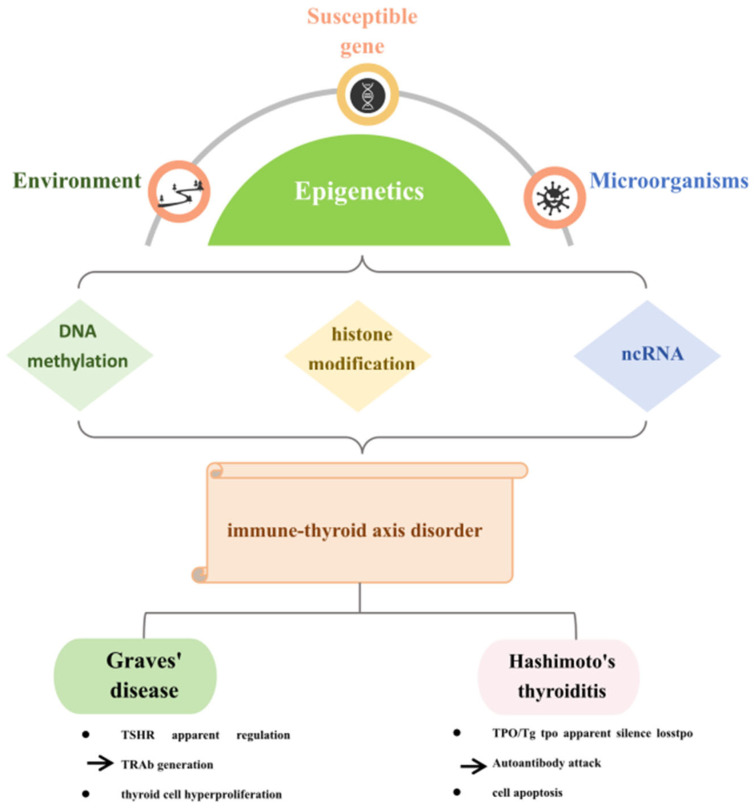
Pathogenesis of autoimmune thyroid disease. This image was created with the Microsoft PowerPoint (Microsoft Corp., Redmond, WA, USA) software version 2019. This diagram illustrates the multifactorial contributors to immune-thyroid axis disorders, emphasizing the complex interplay between genetic susceptibility, environmental factors, and microorganisms. It highlights that epigenetic mechanisms—specifically DNA methylation, histone modification, and non-coding RNA (ncRNA)—mediate these interactions. Disruptions in these epigenetic modifications can impair the immune-thyroid axis, leading to conditions such as Graves’ disease and Hashimoto’s thyroiditis. Graves’ disease is characterized by dysregulation of the TSH receptor (TSHR), production of thyroid-stimulating immunoglobulins (TRAb), and hyperproliferation of thyroid cells. In contrast, Hashimoto’s thyroiditis is characterized by T-cell-mediated autoimmune destruction of thyroid follicular cells. This leads to impaired production of thyroid peroxidase (TPO) and thyroglobulin (Tg), and is associated with the presence of autoantibodies against these antigens (TPOAb and TgAb). The process ultimately results in widespread apoptosis and hypothyroidism.

To ensure a systematic and reliable literature review, this study conducted a thorough search for relevant publications from 2010 to the present in databases such as PubMed, Web of Science, and Embase. The search terms focused on two primary categories: epigenetics (including DNA methylation, histone modifications, and non-coding RNAs) and AITD (such as autoimmune thyroiditis, GD, and HT). After removing duplicates and an initial screening, a total of 137 high-quality articles were included. The selection process followed specific inclusion criteria: studies had to provide evidence from at least two of the following categories: clinical samples, animal models, cellular validations, or sequencing validations. Research based solely on a single type of evidence or unvalidated bioinformatics analyses was excluded. Ultimately, 84 studies meeting the criteria were included; among these, 43 studies provided evidence from two levels, 30 studies included three levels, and 4 studies encompassed all four levels. Regarding specific evidence types, all studies included molecular data, 63 studies featured cellular experiments, 12 studies utilized animal models, and 76 studies reported human-related data. By constructing a multi-tiered evidence framework, this study lays a methodological foundation for a deeper exploration of the epigenetic mechanisms underlying autoimmune thyroid diseases. This review synthesizes current knowledge on epigenetic regulation in AITD pathogenesis, highlights its clinical implications, and proposes novel diagnostic and therapeutic strategies, as shown in Figure 1.

## 2. The Role of DNA Methylation in AITD

DNA methylation is a fundamental epigenetic mechanism that dynamically regulates gene expression through covalently modifying cytosine residues. The crucial biochemical process involves DNA methyltransferases (DNMTs) mediating the transfer of methyl groups from S-adenosylmethionine (SAM) to specific cytosine residues, predominantly at CpG dinucleotides. In mammals, DNA methylation primarily targets cytosine bases within CpG dinucleotides, with DNMTs catalyzing the addition of methyl group to C5 position of cytosine, creating 5-methylcytosine (5-mC) [22]. DNA methylation operates through two primary modes: de novo methylation, which establishes new epigenetic patterns, and maintenance methylation, which preserves existing methylation signatures during cellular replication. DNMT1, in conjunction with UHRF1, ensures the faithful replication of existing methylation marks during DNA replication, while DNMT3A and DNMT3B are responsible for de novo methylation, introducing marks at previously unmethylated CpG sites. Notably, methyl-CpG binding domain proteins (e.g., MBD2, MeCP2) recognize methylated DNA and modulate transcriptional via two main mechanisms: recruiting chromatin-remodeling and sterically hindering transcription factor binding [23]. Through these actions, DNA methylation regulates gene expression by shaping chromatin accessibility, chromatin architecture, and replication fidelity. Accumulating evidence implicates DNA methylation in both normal thyroid physiology and AITD pathogenesis [24].

Emerging genome-wide methylation studies have revealed disease-specific DNA methylation abnormalities in GD cohorts, a major AITD subtype. More than 200 differentially methylated genomic regions have been reported, spanning hypermethylated promoters and hypomethylated regulatory elements, which may promote thyroid autoimmunity by epigenetically dysregulating immune cell function and the expression of immune activation genes [23]. Pathogenic methylation aberrations in key immunoregulatory genes—including *CTLA4*, *CD247*, *PTPN22*, *DNMT1*, and *MECP2*—demonstrate robust associations with GD pathogenesis [23,25,26]. Limbach et al. [27] reported over 300 differentially methylated sites in CD4^+^ T cells and in excess of 3000 differentially methylated sites in CD8^+^ T cells derived from GD patients, largely affecting T cell signaling pathways. Guo et al. [28] observed genome-wide hypomethylation with reduced *DNMT1* expression in both T and B lymphocytes from GD patients. These observations align with the core immunopathology of AITD, in which T- and B-lymphocyte infiltration of thyroid tissue drives autoimmune-mediated structural and functional damage [29,30]. Notably, Limbach et al. [27] also detected significant hypermethylation within TSHR intron 1 in GD patients, suggesting thyroid-specific epigenetic dysregulation contributes to AITD pathogenesis. Taken together, dysregulated DNA methylation at disease-relevant loci appears to drive AITD pathogenesis through epigenetic reprogramming of immune homeostasis, lymphocyte activation pathways, effector cell functions, and thyroid autoantigen expression. Epigenetic dysregulation also involves adhesion molecules. Shalaby et al. [19] detected significant promoter hypomethylation of the Intercellular Adhesion Molecule-1 (*ICAM-1*) gene in AITD patients, potentially explaining enhanced leukocyte-endothelial interactions in thyroid tissue. Because promoter hypomethylation typically increases transcription, *ICAM1* hypomethylation likely elevates *ICAM-1* expression and promotes leukocyte recruitment to the thyroid [31,32]. In AITD, *ICAM-1* expressed by thyrocytes recruits lymphocytes and sustains autoimmune inflammation through T cell activation loops [11,33]. Consistent with this mechanism, anti-*ICAM-1* monoclonal antibodies ameliorate disease manifestations and reduce pathogenic autoantibodies and thyroid hormone levels in GD mouse models, supporting a pathogenic role for *ICAM-1* [34]. These data highlight the value of mapping disease-associated epigenetic alterations and suggest that concurrently targeting adhesion molecules and epigenetic drivers may provide synergistic clinical benefit.

Chen et al. [35] further reported promoter hypomethylation of the *CCL5* and *CXCL8* chemokine genes in AITD patients, correlating with elevated transcript levels and likely enhancing lymphocyte homing to the thyroid. Given the inverse relationship between promoter methylation and transcription, these hypomethylation patterns may serve as epigenetic biomarkers for AITD stratification and as actionable targets for epigenetically informed diagnostics and therapeutics [35]. Beyond chemokine genes, Kyrgios et al. [25] identified *IL2RA* promoter hypomethylation in GD patients that correlates with transcriptional upregulation. As *IL2RA* (CD25) modulates Treg function, this finding may help explain impaired immune tolerance in AITD. Additionally, Jiang et al. [36] found significant hypomethylation in the promoters of *IL17*, *IL21*, and *IL22* in GD patients. Key CpG sites exhibited diagnostic potential (AUC > 0.7) and correlated with TRAb and FT4 levels, emphasizing Th17 epigenetic dysregulation as a crucial feature and potential biomarker for GD.

Moreover, standard GD therapies—antithyroid drugs or radioiodine—can normalize aberrant methylation patterns while improving clinical hyperthyroidism, and this epigenetic normalization may support sustained remission by restoring immune homeostasis [37]. In a study by Xu and colleagues, DNA methylation at multiple CpG sites within the *IL-10* gene was significantly elevated in patients with GD, and methylation at specific sites correlated positively with clinical measures such as FT3 and TRAb levels, highlighting the potential utility of *IL-10* methylation patterns for GD diagnosis, risk stratification, and monitoring [38]. Liu et al. [39] identified *CLTA*, *EDIL3*, *HAPLN1*, and *HIP1* as markedly upregulated in HT. These genes exhibit low methylation, recurrent mutations, and copy-number variations and are associated with poor survival in TC, suggesting their value as diagnostic biomarkers and potential therapeutic targets.

These observations underscore the therapeutic relevance of pathogenic methylation signatures and position epigenetic editing as a promising strategy for disease modification in AITD. DNA methyltransferase inhibitors and locus-specific epigenetic editing approaches could, in principle, reverse autoimmune-permissive epigenetic states in a locus- and context-dependent manner.

Overall, DNA methylation dysregulation constitutes a core mechanism in AITD, offering mechanistic insight and tangible targets for intervention. Current evidence indicates that pathogenic methylation programs drive disease progression through three interconnected processes: aberrant expression of immune response genes, altered lymphocyte effector functions, and dysregulated thyroid autoantigen presentation. This tripartite model explains how epigenetic changes can simultaneously potentiate systemic autoimmunity and organ-specific pathology. Elucidating DNA methylation dynamics and their interactomes will deepen our understanding of AITD and enable the development of early diagnostic biomarkers, personalized therapeutics, and prognostic stratification tools—addressing critical unmet needs in AITD management (see Table 1).

## 3. The Role of Histone Modification in AITD

In addition to DNA methylation, histone modifications are core epigenetic regulators that encompass diverse post-translational changes—chiefly lysine methylation and acetylation—that remodel chromatin structure and function. This plasticity enables rapid transcriptional reprogramming during lymphocyte activation in AITD. Immune signaling pathways precisely orchestrate these modifications through antagonistic enzyme pairs: histone acetyltransferases (HATs) and deacetylases (HDACs) controlling acetylation whereas histone methyltransferases (HMTs) and demethylases (HDMs) regulate methylation states, thereby shaping transcriptional programs in lymphocytes. By reconfiguring nucleosome architecture and chromatin compaction, these modifications alter DNA accessibility to the transcriptional machinery, directly influencing transcription factor binding kinetics at target loci and enabling the spatiotemporal control of gene regulatory networks [48,49]. In AITD, pathogenic histone landscapes disrupt the transcriptional control of inflammatory mediators, promoting immune dysregulation and pathological overexpression of effector molecules that drive disease progression [50]. Histone acetylation is a fundamental post-translational modification predominantly targeting epsilon-amino groups of conserved lysine residues within the basic N-terminal tails of histones. HATs transfer acetyl groups to these lysine, neutralizing their positive charge, weakening histone-DNA electrostatic interactions, and thereby increasing chromatin accessibility to facilitate transcription factor binding and transcriptional activation [51]. Acetylation also creates binding platforms for transcriptional complexes and cooperates with other epigenetic marks within the histone-code framework to regulate processes such as transcriptional activation and DNA replication; conversely, its dysregulation has been implicated in AITD pathogenesis [52]. Consistent with this, Yan et al. [53] reported significantly reduced global H4 acetylation in PBMCs from GD patients compared with healthy controls, and Stephanie et al. [52] observed significantly reduced H3 acetylation in both GD and HT patients versus healthy controls, with decreases exceeding physiological variation. Together, these findings support histone-modification dysregulation, particularly hypoacetylation, as a pathogenic mechanism in GD.

Converging evidence indicates key histone-modifying enzymes shape chromatin architecture and gene expression, thereby influencing cell growth, differentiation, apoptosis, and angiogenesis [52]. HDAC remove acetyl groups from histone lysine, restore positive charge, promote chromatin compaction, and repress transcription [54]. Inhibiting HDAC activity induces histone hyperacetylation and chromatin relaxation, which generally favors transcriptional activation [55]. In perinatal hypothyroid mice, Suset et al. [56] reported that HDAC3-selective inhibitor RGFP966 increases histone acetylation at thyroid hormone receptor (TR) loci in the cerebellum. In a murine model of experimental autoimmune thyroiditis (EAT), Zhang et al. [57] demonstrated that HDAC6 inhibition ameliorates pathology by limiting Th17 cell differentiation. Together, these findings highlight histone-modifying enzymes as clinically translatable epigenetic targets for AITD. Given the established clinical utility of HDAC inhibitors (HDAC) in oncology and hematology, their potential in AITD warrants prospective clinical investigation.

Histone methylation, another fundamental chromatin modification, occurs predominantly on the N-terminal lysine and arginine residues of histones H3 and H4. Histone methyltransferases (HMTs) catalyze this reaction using S-adenosyl-L-methionine (SAM) as the methyl donor, while histone demethylases (HDMs) remove these marks [55]. Methylation can exist as mono-, di-, or trimethyl states, each associated with distinct effects on transcriptional activation or repression within immune gene networks [58]. Beyond transcriptional control, histone methylation helps maintain heterochromatin, regulate genomic imprinting, facilitate DNA repair, and coordinate gene programs essential for immune function. Dysregulated histone methylation has been implicated in AITD pathogenesis [59]. Ni et al. [60] reported significantly reduced global *H3K9* and *H3K4* methylation in PBMCs from GD patients compared with healthy controls, and Limbach et al. [27] detected significant global reduction of *H3K4me3* marks at promoter regions in CD4^+^ and CD8^+^ T cells from GD patients. In thyroid-associated ophthalmopathy (TAO), research led by Xu et al. [61] identified lysine-specific demethylase 1 (LSD1) as a key driver of adipogenesis. Their study demonstrated that LSD1 activates adipogenic genes by removing H3K9me2 marks. Furthermore, targeting LSD1 inhibited fat cell formation, suggesting its potential as a therapeutic target for treating TAO.

The growing clinical relevance of histone methylation has spurred development of therapeutics targeting HMTs and HDMs for immune-mediated disorders [62,63]. Among HMT inhibitors, agents directed at EZH2 show particular promise: Tazemetostat (EPZ-6438), an FDA-approved selective EZH2 inhibitor, suppresses histone H3 lysine 27 trimethylation (*H3K27me3*) and has demonstrated efficacy in certain follicular lymphomas and epithelioid sarcomas, while valemetostat has shown clinical benefit in adult T-cell leukemia/lymphoma [52]. By modulating lymphocyte histone methylation, such agents may correct aberrant immune responses that drive AITD. Elucidating histone-modification dysregulation in AITD will deepen mechanistic understanding and inform targeted, epigenetics-based therapies with greater precision for disease management (see Table 2).

## 4. The Role of Non-Coding RNA in AITD

Non-coding RNAs (ncRNAs) are a diverse group of transcripts that regulate immune gene expression at both the transcriptional and translational levels [66]. They include microRNAs (miRNAs), long non-coding RNAs (lncRNAs), and circular RNAs (circRNAs), all of which are integral to autoimmune pathways. MiRNAs repress target genes by binding messenger RNAs (mRNAs) and promoting transcript degradation or inhibiting translation [67]. LncRNAs and circRNAs, increasingly recognized as epigenetic regulators, modulate gene expression through multifaceted interactions with chromatin, RNA transcripts, and protein complexes, thereby forming multidimensional networks that fine-tune transcriptional programs in immune cells [68]. Accumulating evidence indicates that ncRNAs play a critical role in the pathogenesis of AITD [69,70,71].

### 4.1. Emerging Role of microRNA in AITD

As 20–24 nt single-stranded ncRNAs, miRNAs silence target genes post-transcriptionally by destabilizing mRNAs and repressing translation, thereby shaping immune responses [72]. The biogenesis of miRNA initiates with the RNA polymerase II-driven transcription of primary miRNA transcripts (pri-miRNAs) within the nucleus, followed by Drosha-mediated cleavage to produce precursor miRNAs (pre-miRNAs). Subsequent to this, Exportin-5 facilitates the transport of pre-miRNAs to the cytoplasm, where they are further processed into mature miRNAs by the Dicer enzyme in conjunction with TAR RNA-binding protein 2 (TRBP2). These mature miRNAs are then incorporated into the RNA-induced silencing complex (RISC), where they direct Argonaut proteins to complementary sequences within the 3′UTR of target mRNAs, resulting in either mRNA cleavage or translational repression [73]. MiRNAs play crucial roles in regulating essential cellular processes such as proliferation, differentiation, and apoptosis, and are pivotal mediators in the pathogenesis of autoimmune and inflammatory diseases [74]. MiRNAs maintain cellular homeostasis under physiological conditions and modulate disease pathogenesis [75,76,77].

Aberrant miRNA expression has been documented in serum, plasma, and PBMC samples from AITD patients [78,79,80,81]. Dysregulated miRNAs reported in AITD include miR-101-3p, miR-660-5p, miR-144-3p, miR-762, miR-22, miR-375, and miR-451 [80,82,83]. Serum levels of miR-22, miR-375, and miR-451 are consistently elevated in both HT and GD patients, supporting their potential as biomarkers [84]. miR-346 correlates positively with GD relapse [85,86]. In GD, remission is associated with higher miR-23b-5p and miR-92a-3p and lower let-7g-3p and miR-339-5p compared with non-remission, suggesting utility for prognosis and refractoriness prediction [81]. MiRNAs contribute to AITD progression through integrated mechanisms: they regulate thyroid hormone production and thyrocyte proliferation, apoptosis, differentiation, and senescence, while also modulating systemic immune homeostasis via multiple signaling pathways [83]. In orbital fibroblasts from GO patients, miR-146a bidirectionally controls extracellular matrix components: its upregulation reduces hyaluronic acid and collagen I production, whereas its depletion increases both, thereby exacerbating glycosaminoglycan accumulation and collagen deposition [87]. Co-dysregulation of miR-146a and miR-155 promotes GO fibroblast expansion by releasing proliferation constraints, mechanistically linking these miRNAs to fibrotic pathogenesis [88]. Collectively, these findings not only map the miRNA regulatory circuitry in AITD but also provide a conceptual framework for dissecting molecular disease mechanisms.

The study by Yu et al. [89] identifies miR-182-5p as highly expressed in orbital connective tissue and CD34^+^ fibroblasts from patients with TED. miR-182-5p is regulated by the *IL-6/STAT3* signaling pathway and promotes fibroblast proliferation, migration, fibrosis, and resistance to apoptosis by targeting Smad7, indicating its potential as a therapeutic target for TED. He et al. [90] examine miR-5572 in orbital fibroblasts from TED patients, showing that it is downregulated relative to controls and that loss of miR-5572 increases *COL1A1* expression by relieving repression of its direct target, *F2RL2*. Liu et al. [91] demonstrate that miR-320a regulates orbital fibroblast activation, fibrosis, and oxidative stress in TED through its interaction with *PRDX3*, suggesting that therapeutic modulation of the miR-320a/*PRDX3* axis may mitigate oxidative stress and fibrosis.

MiRNA-based therapeutics generally follow two complementary strategies [92]. Firstly, miRNA inhibition uses antagonists (e.g., anti-mis, LNAs, or antagomirs) to neutralize dysregulated miRNAs and restore normal regulation [93]. Designed oligonucleotides achieve potent miRNA inhibition through high-affinity binding followed by target sequestration or catalytic degradation [94]. Concurrently, small-molecule miRNA inhibitors are also being developed. Second, miRNA replacement restores pathologically reduced miRNAs using synthetic mimics of physiologically abundant, disease-depleted species [92,95]. For example, engineered miR-34a therapeutics have shown anti-proliferative activity in oncology, with a phase 1 trial reporting reduced tumor burden in non–small cell lung cancer (NCT01829971), illustrating the promise of replacement strategies [96]. Although miRNA therapies for AITD are still at an early stage, disease-specific miRNA signatures provide a rational basis for therapeutic development and show strong translational potential.

### 4.2. Emerging Role of lncRNA in AITD

LncRNAs are transcripts longer than 200 nucleotides that regulate immune gene networks through transcriptional and post-transcriptional mechanisms [97]. They are synthesized primarily by RNA polymerase II as primary transcripts (pri-lncRNAs) that undergo canonical processing (capping, splicing, and often polyadenylation) to generate mature molecules. Their functions are tightly linked to subcellular localization: nuclear lncRNAs govern transcriptional programs, whereas cytoplasmic lncRNAs predominantly mediate post-transcriptional immune regulation [98]. In the nucleus, lncRNAs (i) scaffold ribonucleoprotein complexes, (ii) activate or repress transcription of immune-relevant genes, (iii) regulate mRNA processing and stability, and (iv) recruit chromatin-modifying enzymes [99]. In the cytoplasm, they modulate mRNA translation and decay, influence protein localization and activation, and act as competitive endogenous RNAs that sponge miRNAs to shape intercellular immune signaling [100]. Through such multi-layered control, lncRNAs help maintain cellular homeostasis but can also drive disease pathogenesis when dysregulated [101,102,103,104]. Their consistent dysregulation across diseases positions lncRNAs as promising diagnostic and prognostic biomarkers [105].

Accumulating evidence implicates lncRNAs in the pathophysiology of AITD [106]. RUNX1-IT1 contributes to thyroid cell dysfunction by modulating immunomodulatory and pro-inflammatory pathways [98]. Zhao et al. [107] demonstrated that LINC01061 sequesters miR-612, thereby upregulating BRD4. This axis enhances thyrocyte proliferation, drives inflammatory responses and inflammasome activation, while concurrently suppressing apoptosis. Christensen et al. [108] identified a correlation between lncRNA Heg and TRAb/CD14 mRNA expression in GD monocytes, and in vitro Hegg suppresses CD14 transcription in immune cells. Peng et al. [109] confirmed elevated lncRNA-IFNG-AS1 expression in HT patients, associated with Th1 cell polarization and enhanced IFN-γ production. LncRNA-IFNG-AS1 modulates *IFN-γ* expression in CD4^+^ T cells, potentially propagating HT autoimmunity. Li et al. [110] implicated lncRNA-PVT1 in HT via interaction with miR-146a that contributes to Th17/Treg imbalance, and proposed combined detection of PVT1 and miR-146a as a high-value diagnostic biomarker pair for HT. Shi et al. [106] study reveals that lncRNA ENST00000581911 is significantly upregulated in the extraocular muscles (EOM) of TED patients and may contribute to EOM remodeling by modulating inflammation, muscle contraction, and cell proliferation, highlighting it as a potential therapeutic target for TED.

Together, these studies identify lncRNAs as key molecular regulators of AITD pathogenesis and highlight their diagnostic potential in autoimmune thyroid disorders. Nonetheless, technical hurdles—especially low evolutionary conservation and low expression levels—still limit comprehensive mapping of lncRNA-centered immune networks in autoimmunity. Clarifying lncRNA mechanisms in AITD therefore remains a priority with tangible therapeutic implications.

### 4.3. Emerging Role of Other ncRNA in AITD

CircRNAs are covalently closed non-coding transcripts that have emerged as key regulatory molecules in biomedical research. Their circular conformation confers exceptional stability, allowing circRNAs to persist and modulate inflammatory pathways in autoimmune disorders [111]. Beyond acting as molecular sponges that sequester miRNAs through multiple binding sites and thereby influence downstream gene expression [112], circRNAs also interact with RNA-binding proteins (RBPs) to alter protein activity and reshape transcriptional programs [113]. Advances in high-throughput sequencing technologies have greatly expanded the known circRNA repertoire, clarified their biological functions, and underscored their regulatory significance in AITD pathogenesis [114].

In HT, upregulated circRNAs act as ceRNAs and engage immune-relevant pathways, including chemokine signaling, HIF-1 activation, and FoxO regulation [115]. Consistent with this mechanism, several studies show that circRNAs function as potent miRNA sponges, thereby derepressing target mRNAs and modulating HT immunopathology [116]. A representative example is circPHF16, which sequesters miR-378a-3p and derepresses *IL6ST* (encoding gp130), leading to amplified gp130–STAT3 signaling and enhanced IL-17A-driven thyroid autoimmunity [117]. In GD, increased circPHF16 expression may similarly enable miR-378a-3p sequestration and IL6ST depression, thereby intensifying T cell-derived IL-17A responses, implicating circPHF16 as both a mechanistic contributor to GD immunopathology and a candidate diagnostic and therapeutic target [118]. Collectively, these data broaden current understanding of circRNA function and provide mechanistic insights into autoimmune thyroid disorders.

Small interfering RNAs (siRNAs) are double-stranded non-coding RNAs of approximately 20–25 nucleotides that mediate sequence-specific gene silencing and represent a versatile RNA interference (RNAi) modality [119]. As central RNAi effectors, siRNAs guide Argonaute-containing RNA-induced silencing complexes (RISCs) to complementary mRNAs, leading to Endo nucleolytic cleavage and suppression of target gene expression [120]. In GD, pathogenic autoantibodies hyperactivate the TSHR on thyrocytes, driving sustained thyroid dysfunction [88]. This disease mechanism highlights TSHR-directed siRNA as a rational therapeutic strategy, and administration of TSHR-targeting siRNA has improved disease phenotypes in experimental GD models [34]. More broadly, siRNA offers a precise tool for functional genomics and therapeutic pathway modulation, particularly when computationally optimized to enhance efficacy and specificity [121]. Although AITD-focused siRNA studies remain preliminary, their targeting precision supports further development for next-generation GD therapeutics.

Together, current evidence establishes non-coding RNAs as functionally significant contributors to AITD immunopathology and as tractable nodes for precision intervention. miRNAs operate as master regulators and promising therapeutic targets; lncRNAs have validated roles in AITD; circRNAs—exemplified by circPHF16—are emerging as important modulators; and siRNA provides a programmable platform for selective gene knockdown in GD. These insights not only refine our understanding of immune dysregulation in AITD but also guide the rational design of ncRNA-based therapies. (see Table 3).

## 5. Conclusions and Perspectives

**Figure 2:**
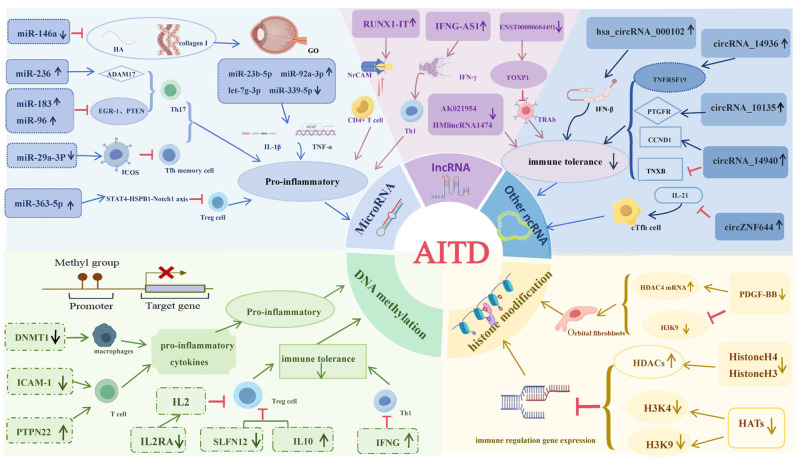
Mechanism of epigenetics regulation in the pathogenesis of AITD. Created in BioRender Smith, d. (2025). BioRender.com/c248457. Non-coding RNAs (miRNAs, lncRNAs, circRNAs), DNA methylation, and histone modifications converge to shape inflammatory signaling and immune tolerance in AITD. MiRNAs (e.g., miR-146a, miR-236, miR-183/96) modulate pathways in Th17, Tfh/cTfh, and Treg cells. LncRNAs (e.g., *RUNX1-IT*, *IFNG-AS1*) influence Th1 polarization, CD4^+^ T-cell activation, and *IFN-γ* production. CircRNAs (e.g., hsa_circRNA_000102, circRNA_14936) have been linked to Tfh/cTfh-mediated immune tolerance. DNA methylation (e.g., *DNMT1* activity; methylation changes at *ICAM-1* and *PTPN22*) and histone marks (e.g., *H3K4me3*, *H3K9me3*) alter the expression of inflammation-related genes, collectively forming regulatory networks that contribute to AITD pathophysiology.

In summary, epigenetic regulation offers a cohesive framework for understanding immune dysregulation in AITD. Key insights reveal that changes in DNA methylation, histone modifications, and non-coding RNAs are linked to enhanced leukocyte recruitment and sustained inflammation. Translational opportunities include rigorously validated circulating miRNA panels and methylation signatures for diagnostic and prognostic purposes, as well as selective inhibitors of histone-modifying enzymes and RNA-based therapies, as shown in Figure 2. However, significant knowledge gaps remain, particularly regarding cell-type specificity and causality. Future research should prioritize multi-layered, longitudinal investigations using advanced techniques like single-cell epigenomic profiling and CRISPR-based methods to establish mechanisms and improve clinical outcomes through biomarker-guided interventions.

The emerging field of epigenetic therapeutics offers promising avenues for direct intervention in AITD. Evidence from experimental models indicates that specific epigenetic modifications can be therapeutically targeted. For instance, HDAC6-specific inhibition has been shown to alleviate HT by suppressing Th17 cell differentiation, highlighting the potential of targeting histone modifications. In models of GD, the application of TSHR-targeting siRNA successfully ameliorated disease phenotypes, providing proof of concept for RNA interference-based strategies. Furthermore, modulation of the miR-320a/*PRDX3* axis has been demonstrated to reduce oxidative stress and fibrotic changes in thyroid eye disease fibroblasts, suggesting the viability of miRNA-based approaches for managing AITD complications. Collectively, these findings map a path from fundamental epigenetic discovery to targeted therapeutic applications, positioning epigenetic modulation as a compelling frontier for future AITD treatment strategies.

## Figures and Tables

**Table 1:** Mechanisms and Clinical Significance of DNA methylation Dysregulation in AITD.

Disease	Sample Source	Disorder	Function	Effects in AITD
HT [25]	PBMC	*PTPN22* methylation increased	Gene underexpression disrupts immune regulation, leading to loss of immune tolerance, immune dysfunction, and excessive inflammation.	Pathogenesis
GD [40]	T cells	*IL2RA* methylation significantly decreased	Treg and immune tolerance, it disrupts the balance of the immune system, increasing the risk of GD in adolescents.	Clinic treatment
GD\HT [35]	Thyroid tissue	*CCL5* and *CXCL8* methylation decreased	May serve as epigenetic markers and bioindicators of AITD.	Disease markers
GD\HT [41]	PBMC	*ICAM-1* methylation decreased	High *ICAM-1* gene expression exacerbates the immune response in AITD.	Clinic treatment
GD\HT [42]	PBMC	*CTLA4* methylation decreased	Gene overexpression inhibits T cell activation, affecting immune regulation.	Pathogenesis
GD\HT [43]	PBMC	*IL6* methylation levels decreased	Gene overexpression worsens disease progression.	Pathogenesis
GD\HT [44]	PBMC	*IFNG* methylation increased	*IFNG* is related to the pathogenesis and prognosis of AITD, helpful in distinguishing refractory GD patients.	Disease markers
GO [45]	Fibroblasts	*HAS* significantly increased methylation	Decreased *HAS* gene expression reduces inflammation, alleviating AITD.	Clinic treatment
GD [46]	PBMC	*IL10* methylation increased	Reduced production of *IL10* affects immune cell proliferation, reducing inflammation.	Clinic treatment
HT [47]	PBMC	*SLFN12* methylation decreased	High expression of *SLFN12* disrupts immune tolerance, enhances immune activation, and promotes the development of AITD.	Pathogenesis

Abbreviations: PBMC = Peripheral blood mononuclear cells; HT = Hashimoto’ s Thyroiditis; GD = Graves’ Disease; GO = Graves’ ophthalmopathy; PBMC = peripheral blood mononuclear cells; *PTPN22* = Protein Tyrosine Phosphatase Non-Receptor Type 22; *IL2RA* = Interleukin-2 Receptor Alpha; *CCL5* = C-C Motif Chemokine Ligand 5; *CXCL8* = C-X-C Motif Chemokine Ligand 8; *ICAM-1* = Intercellular Adhesion Molecule-1; *CTLA4* = Cytotoxic T-Lymphocyte-Associated Protein 4; *IL6* = Interleukin-6; *IFNG* = Interferon Gamma; *HAS* = Hyaluronan Synthase; *SLFN12* = schlafen family member 12.

**Table 2:** Mechanisms and Clinical Significance of Histone Modifications in AITD.

Disease	Sample Source	Disorder	Function	Effect in AITD
GD\HT [52]	PBMC	*Histone H3* significantly decreased acetylation level	Uncontrolled immune response leading to intensified immune system attack on the thyroid, resulting in thyroid dysfunction	Disease markers, target therapy
GD [53]	PBMC	*Histone H4* significantly decreased acetylation level	Inhibition of genes related to immune cell function, further exacerbating immune response and inflammation	Target therapy
GO [64]	Orbital fibroblasts	*PDGF-BB* significantly increased HDAC4 mRNA expression, low *H3K9* acetylation	Stimulated induction of orbital fibroblast activation plays a key role in the pathogenesis of GO	Target therapy
GD [59]	PBMC	*H3K4*, *H3K9* significantly decreased histone methylation	Loosening chromatin structure, allowing the activation and expression of inhibited genes	Pathogenesis
HT [65]	Thyroid tissue	Enriched *H3K4me3* increase	Upregulation of genes related to immune, inflammation, and thyroid function, further intensifying self-immune and inflammatory responses	Pathogenesis

Abbreviations: PBMC = Peripheral blood mononuclear cells; HT = Hashimotos’ Thyroiditis; GD = Graves’ Disease; GO = Graves’ ophthalmopathy; PDGF-BB = Platelet-Derived Growth Factor-BB; HDAC4 = Histone Deacetylase 4; *H3K9* = Histone H3 Lysine 9; *H3K4me3* = Histone H3 Lysine 4 trimethylation.

**Table 3:** Differential Expression and Clinical Significance of Non-Coding RNAs in AITD.

Disease	Sample Source	Expression	Function	Effect in AITD
GD [121]	Orbital Fibroblasts	Upregulation of miR-146a	Significant regulatory function in orbital fibroblasts	Clinic treatment
GD [84]	PBMC	Upregulation of miR-23b-5p and miR-92a-3p, downregulation of let-7g-3p and miR-339-5p	Potential biological targets for predicting GD treatment prognosis and disease resistance	Clinic treatment
GD\HT [122]	Ciliary Tissue	Upregulation of miR-21-5p, miR-146b-3p, miR-5571-3p, and miR-6503-3p	Upregulated expression positively correlated with ciliary growth in thyroid cells, indicating potential therapeutic targets for AITD	Clinic treatment
GD [123]	Tregs	Upregulation of miR-30a-5p, miR-181a, miR-636	Significant reduction in the number and immune suppressive function of Tregs in initial GD patients	Clinic treatment
GD [124]	Peripheral T Cells	Upregulation of miR-183, miR-96	Increased production of pathogenic cytokines in Th17 cells during development	Pathogenesis
GD [125]	Tfh Memory Cells	Downregulation of miR-29a-3p	Limitation of circulating Tfh memory cell response in GD patients	Clinic treatment
GD [82]	Serum	Upregulation of miR-210, downregulation of miR-182, miR-155, miR-146a	Potential new biomarkers for diagnosing GD	Disease markers
GD [126]	Serum	Upregulation of miR-346, downregulation of TRAb	Predictive factors for Graves’ Disease recurrence	Disease markers
GD [87]	Plasma	Downregulation of miR-144-3p, upregulation of miR-762	Biomarkers for GD diagnosis	Disease markers
GD [127]	Thyroid Tissue	Downregulation of miR-21-5b, miR-19-1538, hsa-miR-182-5p; upregulation of 19-15038, hsa-miR-27a-3p, miR-Let7d-5p	Biomarkers for predicting GD recurrence or severity	Disease markers
GD [128]	Serum Samples	Upregulation of miR-19a, miR-143	Biomarkers for development risk and/or severity	Disease markers
GD [129]	Peripheral Treg Cells	Upregulation of miR-363-5p	Inhibiting proliferation, differentiation, and function of Treg cells through STAT4-HSPB1-Notch1 axis	Clinic treatment
GD [83]	PBMC	Downregulation of miR-154-3p, miR-376b-3p, miR-431-3p	Potential new biomarkers for GD and therapeutic targets	Clinic treatment
GD [130]	CD4^+^ T cells and CD8^+^ T cells	Downregulation of miR-200a-3p and miR-200a-5p in CD4^+^ T cells and CD8^+^ T cells; downregulation of miR-155-5p and miR-155-3p in CD8^+^ T cells	Novel biomarkers for GD	Disease markers
HT [131]	Serum	Upregulation of miR-146a, miR-142, miR-301	Prognostic biomarkers and Clinic treatment	Disease markers
HT [132]	PBMC	Upregulation of microRNA-326	Potential molecular mechanism of miR-326 targeting ADAM17 to promote Th17 cell proliferation in HT patients in vitro	Pathogenesis
HT [133]	T cells	Downregulation of miR-29a-3p	Prognostic biomarkers and therapeutic targets	Disease markers
GD [134]	CD4^+^ T cells	Significant reduction in expression of NONHSAT093153.2, NONHSAT118924.2, NONHSAT209004.1	Potential biomarkers for recurrent GD	Disease markers
GD [135]	PBMC	Significant downregulation of ENST00000604491	Potential biomarker for diagnosing GD through regulation of FOXP1 in Treg cell modulation	Disease markers
GD [135]	PBMC	Decreased expression of AK021954, AB075506, HMlincRNA1474	Potential new biomarkers for GD and therapeutic targets	Clinic treatment
GD [99]	CD4^+^ T cells	Most significant upregulation of *RUNX1-IT1* and *NrCAM*	Provides new research directions for clinical treatment of GD	Clinic treatment
HT [108]	Th1 Cells	Increased expression of *IFNG-AS1*	Enhanced expression contributes to Th1 cell response in HT patients and may be involved in the pathogenesis of HT	Disease markers
HT [115]	PBMC	significantly increased expression of hsa_circ_0089172	Potential diagnostic biomarker for HT	Disease markers
GD [116]	Serum Exosomes	significantly increased expression of hsa_circRNA_000102	Provides new insights into the pathogenesis and treatment of GD	Pathogenesis
GD/GO [136]	Orbital Fat/Connective Tissue	Increased expression of circRNA_14936 and circRNA_14940, decreased expression of circRNA_12367	Differential expression of circRNAs may provide new insights into the pathogenesis of TAO	Pathogenesis
GD [117]	PBMC	Upregulation of circPHF16	Potential target for developing new strategies for the diagnosis and treatment of GD, providing a theoretical basis for clinical treatment methods for GD	Clinic treatment
GD [137]	PBMC	Upregulation of circ*ZNF644*	Key feature of GD development, potential diagnostic biomarker	Disease markers

Abbreviations: PBMC = Peripheral blood mononuclear cells; HT = Hashimoto’ s Thyroiditis; GD = Graves’ Disease; GO = Graves’ ophthalmopathy; *RUNX1-IT1* = RUNX1 Intronic Transcript 1; *IFNG-AS1 =* IFNG Antisense RNA 1; *ADAM17* = Disintegrin and metalloproteinase domain-containing protein 17.

## Data Availability

Not applicable.

## Abbreviations

The following abbreviations are used in this manuscript.

AITD	Autoimmune Thyroid Disease
EAT	Experimental Autoimmune Thyroiditis
EOM	Extraocular Muscles
GD	Graves’ Disease
GO	Graves’ Ophthalmopathy
HT	Hashimoto’s Thyroiditis
TAO	Thyroid-Associated Ophthalmopathy
TED	Thyroid Eye Disease
5-mC	5-Methylcytosine
DNMT	DNA Methyltransferase
DNMT1	DNA Methyltransferase 1
DNMT3A/B	DNA Methyltransferase 3A/3B
HAT	Histone Acetyltransferase
HDAC	Histone Deacetylase
HDACI	Histone Deacetylase Inhibitor
HDM	Histone Demethylase
HMT	Histone Methyltransferase
H3K4me3	Histone H3 Lysine 4 trimethylation
H3K9me2	Histone H3 Lysine 9 dimethylation
H3K27me3	Histone H3 Lysine 27 trimethylation
SAM	S-adenosyl-L-methionine
T4	Thyroxine
Tg	Thyroglobulin
TPO	Thyroid Peroxidase
TR	Thyroid hormone Receptor
TSH	Thyrotropin/Thyroid-Stimulating Hormone
TSHR	TSH Receptor
CCL5	C-C Motif Chemokine Ligand 5
cTfh	Circulating T follicular helper cell
CXCL8	C-X-C Motif Chemokine Ligand 8
ICAM-1	Intercellular Adhesion Molecule-1
IFN-γ	Interferon-gamma
IL-2RA	Interleukin-2 Receptor Alpha Chain
IL-6	Interleukin-6
PBMC	Peripheral Blood Mononuclear Cells
STAT3	Signal Transducer and Activator of Transcription 3
Tfh	T follicular helper cell
Th1	T helper 1 cell
Th17	T helper 17 cell
Treg	Regulatory T cell
TGAb	Thyroglobulin Antibodies
TPOAb	Thyroid Peroxidase Antibodies
TRAb	TSH Receptor Antibodies
ceRNA	Competitive Endogenous RNA
circRNA	Circular RNA
lncRNA	Long non-coding RNA
miRNA	MicroRNA
mRNA	Messenger RNA
ncRNA	Non-coding RNA
pre-miRNA	Precursor microRNA
pri-miRNA	Primary microRNA
RBP	RNA-Binding Protein
RISC	RNA-Induced Silencing Complex
siRNA	Small Interfering RNA
CRISPR	Clustered Regularly Interspaced Short Palindromic Repeats
FDA	U.S. Food and Drug Administration
RNAi	RNA Interference
HAS	Hyaluronan Synthase
PDGF-BB	Platelet-Derived Growth Factor-BB
